# *Asclepiasspeciosa* (Apocynaceae, Asclepiadoideae): a rare or unrecognized alien species in Europe?

**DOI:** 10.3897/phytokeys.121.33573

**Published:** 2019-04-24

**Authors:** Zigmantas Gudžinskas, Lukas Petrulaitis, Egidijus Žalneravičius

**Affiliations:** 1 Nature Research Centre, Institute of Botany, Žaliųjų Ežerų Str. 49, Vilnius LT-08364, Lithuania Nature Research Centre, Institute of Botany Vilnius Lithuania

**Keywords:** alien species, ecology, identification, invasiveness, morphology, naturalization, reproduction

## Abstract

Studies on populations of *Asclepiassyriaca* L. in Lithuania revealed the occurrence of a new alien plant species, the North American native *Asclepiasspeciosa* Torr. (Apocynaceae, Asclepiadoideae), in southern parts of Lithuania – the first report of the latter species in Europe. Interestingly, a thorough analysis of herbarium specimens revealed that *A.speciosa* had first been collected in Lithuania in 1962, but the specimen was misidentified at the time as *A.syriaca*. The newly discovered population of *A.speciosa* occupies mesic grasslands, tall-herb fringe communities and arable field habitats. Sexual reproduction of this species was not recorded; it spreads locally by means of vegetative reproduction. We present here an exhaustive analysis of morphological characteristics and differences between *A.speciosa* and *A.syriaca* and other species of the genus, as well as a key for identification of alien *Asclepias* species in Europe. We predict that the effect of *A.speciosa* on native habitats and communities, and its economic impact, are comparable to those of the highly invasive *A.syriaca*. Although *A.speciosa* currently occurs very rarely as an alien species in Europe, its existence in other regions of Europe is highly probable.

## Introduction

The genus *Asclepias* L. s. str. (Apocynaceae, Asclepiadoideae) includes about 120 species native to the Western Hemisphere. Most of the species are distributed in North America and the Caribbean, ten species occur in South America (Woodson 1954; Wilbur 1976; Endress and Bruyns 2000; Agrawal et al. 2009). However, if a broad concept of the genus *Asclepias* is accepted, it would include ca. 400 species distributed in Africa, West Asia, North and South America (Goyder 2009; Chuba et al. 2017). Several species of the genus *Asclepias* have been registered as aliens in other continents, and some of them are invasive (Ping-Tao et al. 1995; Randall 2007; Botta-Dukát 2008).

In Europe, three alien species of the genus *Asclepias* s. str. had been reported so far: *Asclepiascurassavica* L., *Asclepiasincarnata* L., and *Asclepiassyriaca* L. (Arianoutsou et al. 2010; Pyšek et al. 2017). Another two species, *Asclepiasfruticosa* L. and *Asclepiasphysocarpa* (E.Mey.) Schltr., have also been reported as alien naturalized species in several countries of southern Europe (Greuter and Raus 2000; Knees 2000; Arianoutsou et al. 2010; Haeger et al. 2011); however, these species of African and West Asian origin are frequently considered as members of the genus *Gomphocarpus* R.Br. (*Gomphocarpusfruticosus* (L.) W.T.Aiton and *Gomphocarpusphysocarpus* E.Mey., respectively) (Goyder and Nicholas 2001; Fishbein et al. 2011). About a dozen *Asclepias* species are cultivated as ornamentals in Europe (Knees 2000). The most widespread and invasive species in some South and Central European countries is *A.syriaca* (Botta-Dukát 2008; Konstantinović et al. 2008; Medvecká et al. 2012; Kelemen et al. 2016; Pergl et al. 2016), whereas *A.curassavica* and *A.incarnata* have been recorded as casuals or locally naturalized aliens in various regions of Europe (Verloove 2006; DAISIE 2009; Arianoutsou et al. 2010). Because of invasiveness and significant negative impact on native habitats, as by the provisions of the Regulation of the European Parliament, EU 1143/2014, and of the Council of 22 October 2014 on Invasive Alien Species, *A.syriaca* was included in the list of alien species of European Union concern (EU 2016/1142, EU 2017/1263).

In an attempt to implement the requirements of Regulation EU 1143/2014, we set out with an extensive study of the distribution, habitats, population structure, and impact of *A.syriaca* on native plant communities in Lithuania, because available information on this species was only fragmentary. Over several decades, *A.syriaca* had been reported to occur at several localities in southern Lithuania, and it has been recognized as a naturalized species (Gudžinskas 1998; Gudžinskas et al. 2018). Revisiting and evaluating the current status of populations at all localities recorded in the literature, in herbaria and other archival sources, was among the aims of this study. On a visit to one of the previously reported localities in the district of Alytus (South Lithuania), we noticed significant morphological differences in the plants compared to *A.syriaca* at other sites in Lithuania. Although herbarium specimens from this locality had been identified as *A.syriaca*, further studies showed that this population represented another species of this genus, *Asclepiasspeciosa* Torr. Analysis of checklists of alien species and floristic lists of many European countries (Essl and Rabitsch 2002; Verloove 2006; DAISIE 2009; Arianoutsou et al. 2010; Gederaas et al. 2012; Medvecká et al. 2012; Pyšek et al. 2012; NOBANIS 2018) revealed that *A.speciosa* had neither been reported as naturalized nor as a casual alien species.

The aim of this study was to identify reliable morphological characters for distinguishing *A.syriaca* and *A.speciosa* at the vegetative stage and during flowering, to study habitats of *A.speciosa*, and to evaluate the possibilities of its further spread and invasion.

## Materials and methods

The population of *A.speciosa* was studied in the environs of the village of Liepakojai, Alytus district, South Lithuania (54°28.51'N, 23°40.86'E). Research on morphological characteristics, flowering, and habitats was performed on 26–28 June and 20 September 2018. For comparison of morphological features, we selected a population of *A.syriaca* located in a similar habitat in the village of Meškučiai, Kaišiadorys district (54°44.88'N, 24°10.09'E). In each population, we studied characters of 30 flowering shoots.

Plants for the study were selected randomly from all over the colony, with a distance of at least 2 m between sampled plants. Stems were cut at ground level with garden shears. Stem height was measured from the soil level to the apex with a precision of 1 cm, using a measuring tape. The number of leaf pairs on the stem was counted, including wilted and fallen leaves. In cases where some of the lower leaves were fallen, their number was determined by leaf scars on the stem. Leaf measurements with a precision of 0.1 cm were taken from the leaf that was situated closest to the middle of the stem. The length and width of the leaf blade, length of the petiole, and diameter of the lowermost inflorescence were measured using a ruler. The length of the leaf blade was measured from the leaf base at the junction with the petiole to its tip, whereas the width of the leaf was measured at its widest point. The number of developed inflorescences on the stem was counted. The diameters of the inflorescences were measured at their middle part and the number of flowers was counted.

We studied the herbarium specimens of *Asclepias* species stored at the Herbarium of the Institute of Botany of the Nature Research Centre (BILAS), Vilnius, Lithuania. Specimens collected during this research were also deposited at the same herbarium.

The significance of differences between the studied characters of *A.speciosa* and *A.syriaca* was tested by applying a 2-sample *t*-test. All calculations were performed using PAST 3.20 (Hammer et al. 2001).

## Results

### History of records

The studied herbarium specimens revealed that *A.speciosa* was first collected in Lithuania on 10 August 1962 by M. Vincevičiūtė in the Žuvintas mire (Alytus district, South Lithuania); the specimen was identified by V. Galinis as *A.syriaca* (BILAS, 92607). This record was also mentioned by Galinis (1964), with additional information that the plant had been found about 1 km from the nearest settlement, but had not been found in gardens or ornamental plantings in surrounding settlements. The current state of this population is unknown. Most probably it became extinct because of development of the mire habitat. It should be noted that the specimen collected in 1962 was found only recently among collections transferred from the Lithuanian University of Educology of Vilnius, Lithuania, to BILAS. Based on the information provided by Galinis (1964), the record of *A.speciosa* was erroneously considered as the first record of *A.syriaca* in Lithuania (Gudžinskas 1998).

A new locality for *A.speciosa* was discovered on 24 July 1992 by M. Lapelė in the village of Liepakojai (Alytus district); however, it was also erroneously identified as *A.syriaca* (BILAS, 63179). Just a few days later, *A.speciosa* was collected by Ž. Sinkevičius (BILAS, 87421). More than a decade later, in 2004, a specimen of *A.speciosa* was collected by V. Rašomavičius (BILAS, 76363) and also identified as *A.syriaca*. Therefore, *A.speciosa* has been present in southern Lithuania in the vicinity of the Žuvintas Biosphere Reserve for at least 56 years and at its recently identified locality for at least 26 years.

### Habitats and population size

**Figure 1.**
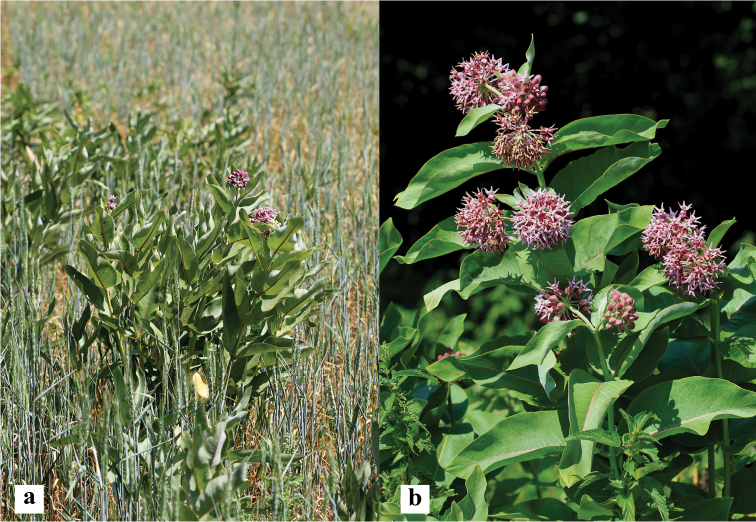
*Asclepiasspeciosa* in a field of winter wheat (**a**) and at the edge of a woodland (**b**).

Galinis (1964) reported an abundant population of *A.speciosa* (originally identified as *A.syriaca*) in the mire of Žuvintas, although the exact size of the population was not given. The label of the herbarium specimen of *A.speciosa* collected by M. Lapelė in the vicinity of the village of Liepakojai indicated that plants were abundant, but the size of the colony was not specified. The herbarium label of the specimen collected in 2004 by V. Rašomavičius noted that this species formed a colony; however, once again, the size of the stand and density of the colony were not recorded. During the present study in 2018, the area occupied by *A.speciosa* in the vicinity of the village of Liepakojai was measured. A dense stand of *A.speciosa* in a mesic meadow and at the edge of a woodland (Fig. 1) occupied an area of 230 m^2^, whereas in a nearby winter wheat field it formed quite a sparse colony over an area of about 280 m^2^. Thus, the total area occupied by *A.speciosa* in 2018 was ca. 510 m^2^.

In the tall herb woodland fringe community (Fig. 1), coverage of *A.speciosa* in 2018 was about 60%, with the most abundant native species being *Urticadioica*, *Aegopodiumpodagraria*, *Chaerophyllumaromaticum*, and *Anthriscussylvestris*. In the mesic grassland, the coverage of *Asclepiasspeciosa* was slightly higher, at about 65%. The most abundant native species in the grassland community were *Geraniumpratense*, *Festucapratensis*, *Phleumpratense*, *Agrostiscapillaris*, *Festucarubra*, *Poapratensis*, and *Medicagofalcata*, though their total coverage was quite small at ca. 25%. In the field with the winter wheat crop, the coverage of *Asclepiasspeciosa* was about 5% (Fig. 1). Apart from *Triticumaestivum*, which covered about 60% of the field, the most abundant species were *Artemisiavulgaris*, *Chenopodiumalbum*, *Cirsiumarvense*, and *Thlaspiarvense*.

### Reproduction

*Asclepiasspeciosa* flowered abundantly in June 2018 in the studied population in the village of Liepakojai; however, no developed fruits were recorded as by September 2018. Therefore, sexual reproduction was probably absent. The colony survives and expands by vegetative renewal, spreading by long rhizomes.

### Morphological characteristics and identification

**Figure 2.**
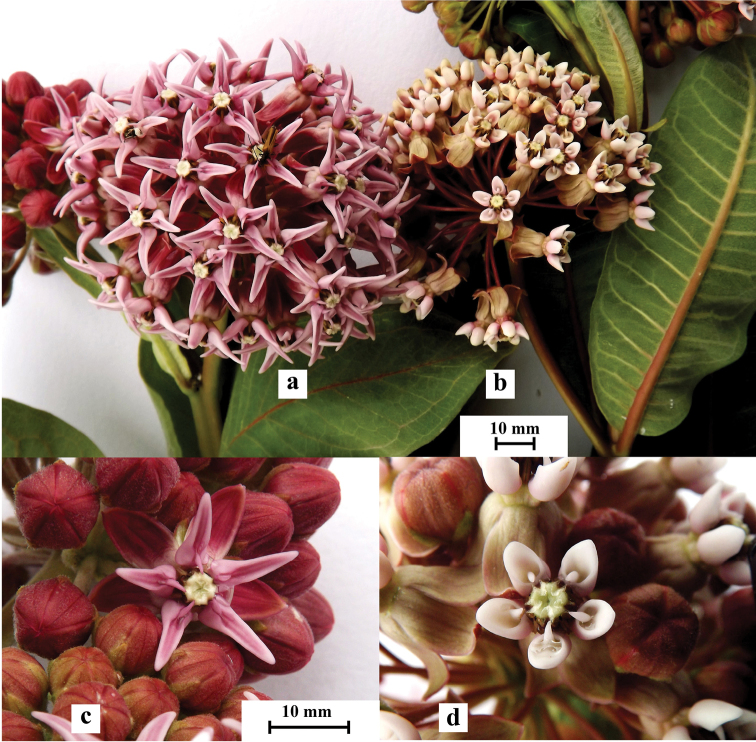
Inflorescences and individual flowers of *Asclepiasspeciosa* (**a, c**) and *Asclepiassyriaca* (**b, d**).

*Ascepiasspeciosa* can most easily be distinguished from the closely related *A.syriaca* at the flowering stage, based on shape and size of flowers (Fig. 2). The most characteristic features of *A.speciosa* are the size and shape of the corona. Hoods of *A.speciosa* are 10–13 mm long, with a prolonged tongue-like apex, and the horns are longer and evidently curved inwards. Hoods of *A.syriaca* are 3.5–5.0 mm long, with a rounded apex, and the horns are short and only slightly bent inwards (Fig. 2). Another reliable character is the indumentum of the pedicels. The pedicels of *A.speciosa* are densely covered with short white hairs, whereas those of *A.syriaca* are glabrous or have only a few such hairs.

Both species also differ in the number of flowers in the inflorescence. Although the diameter of the inflorescence is almost the same in both species (Table 1), those of *A.speciosa* are laxer and contain almost half the number of flowers as those of *A.syriaca* (mean 36.2 ± 7.2 and 61.2 ± 19.5 flowers, respectively).

**Table 1.** Comparison of some morphological characteristics of *Asclepiasspeciosa* and *Asclepiassyriaca.* Mean values are presented with standard deviation and range (minimum and maximum) in parentheses. Different letters denote significant differences of *t*-test (*P* < 0.001) between the same characters.

Character	* Asclepias speciosa *	* Asclepias syriaca *
	Mean ± SD (range)	Mean ± SD (range)
Stem height (cm)	117.6 ± 11.9 (86–134) ^a^	95.7 ± 5.4 (80–105) ^b^
Number of leaf pairs	10.3 ± 0.7 (9–12) ^a^	11.5 ± 0.6 (11–13) ^b^
Length of petiole (cm)	0.9 ± 0.2 (0.5–1.2) ^a^	0.7 ± 0.2 (0.4–1.1) ^b^
Length of leaf blade (cm)	17.1 ± 2.3 (10.0–21.1) ^a^	14.6 ± 1.6 (11.2–18.3) ^b^
Width of leaf blade (cm)	10.1 ± 1.0 (8.1–12.6) ^a^	8.3 ± 0.9 (6.8–10.4) ^b^
Number of inflorescences	3.4 ± 0.8 (2–5) ^a^	3.4 ± 0.7 (2–5) ^a^
Diameter of inflorescence (cm)	7.4 ± 0.3 (6.9–7.9) ^a^	7.1 ± 0.9 (5.2–8.5) ^a^
Number of flowers in inflorescence	36.2 ± 7.2 (22–57) ^a^	61.2 ± 19.5 (30–108) ^b^

**Figure 3.**
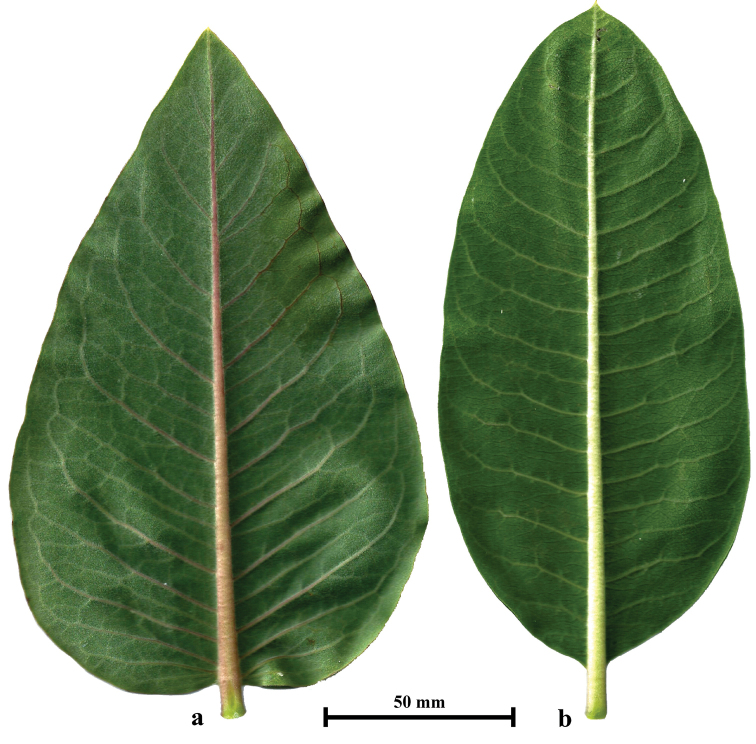
Middle cauline leaves of *Asclepiasspeciosa* (**a**) and *Asclepiassyriaca* (**b**).

Several characteristics of the leaves are reliable for distinguishing *A.speciosa* and *A.syriaca*. The leaf blade of the middle cauline leaves of *A.speciosa* is broadly ovate or elliptical, widest at its slightly cordate base, whereas the leaf blade of *A.syriaca* is ovate or elliptical, with a rounded or slightly cuneate base, and is widest near its middle (Fig. 3). Furthermore, the leaves of *A.speciosa* are significantly longer and wider than those of *A.syriaca*, at least in the studied populations (Table 1). A quite reliable character for distinguishing these two species is leaf venation. Lateral veins in the leaf blades of middle cauline leaves of *A.speciosa* are mainly alternate and ramify from the main vein at a narrow angle, whereas those of *A.syriaca* are usually arranged oppositely and ramify at a wider angle (Fig. 3). Leaf shape and size, as well as leaf venation, can be reliable features for distinguishing the species before or after anthesis, when features of flowers cannot be employed. It should be noted that the leaves of sterile shoots (which were always present in dense stands and at the edge of colonies of both species) significantly differ in shape and size from the leaves of fertile shoots. Usually, the leaves of sterile shoots are much narrower, with more acutely pointed apices.

Although stem height, number of leaf pairs, and length of petiole were statistically different between *A.speciosa* and *A.syriaca* in the studied populations, these features are not reliable for the identification of these species.

Confusion of *A.speciosa* with *A.curassavica*, which is characterized by linear lanceolate or lanceolate leaves and an orange or red corolla and yellow or orange corona, is hardly possible. Confusion is more likely between *A.speciosa* and *A.incarnata*. However, the leaves of *A.incarnata* are narrow lanceolate or lanceolate and the stem is evidently branched. Although the colour of the corona in both species is variable and can be similar, the hoods of *A.incarnata* have a rounded apex and the horns are longer than the hood. Furthermore, *A.incarnata* is a clump-forming plant, whereas *A.speciosa* has long rhizomes and usually forms colonies.

*Asclepiaspurpurascens* L. has not been recorded as having escaped from cultivation in Europe; however, it is occasionally planted in gardens and collections. This species is quite similar to *A.speciosa*, particularly in its vegetative characters. However, the leaf base of *A.purpurascens* is abruptly cuneate, whereas that of *Asclepiasspeciosa* is shallowly cordate or almost rounded, and the petiole is clearly distinct from the leaf blade. *Asclepiaspurpurascens* usually has one terminal and 1–2 additional inflorescences in the upper leaf axils, whereas *A.speciosa* and *A.syriaca*, being more robust plants, have inflorescences in several lower axils as well. Flower characters are the most reliable for distinguishing *A.speciosa*, *A.syriaca*, and *A.purpurascens*. The hoods of *A.purpurascens* are of a similar shape to those of *A.syriaca*, though longer (5–7 mm vs. 3.5–5.0 mm). The hoods of *A.speciosa* are much longer (10–13 mm) than in both other species and differ in shape, having prolonged acute apices rather than short rounded apices.

### Key for the identification of alien *Asclepias* species in Europe

**Table:** 

1	Leaves of reproductive shoots linear lanceolate, narrow lanceolate or lanceolate	**2**
–	Leaves of reproductive shoots elliptic, ovate, or broadly ovate	**3**
2	Stems up to 1 m tall, cymes 10–20-flowered; flower corolla red, orange or yellow, corona yellow or orange	***Asclepiascurassavica* L.**
–	Stems 1.0–1.5 m tall, usually evidently branched; cymes 20–40-flowered, flower corolla bright purple, pink carmine, occasionally white, corona carmine or pink	***Asclepiasincarnata* L.**
3	Leaves of reproductive shoots broadest in basal part, with shallowly cordate or almost rounded base; flower pedicels densely white-tomentose; corolla deep purple or pink, corona pink or pinkish, hoods 10–13 mm long, with a prolonged tongue-like apex	***Asclepiasspeciosa* Torr.**
–	Leaves of reproductive shoots broadest at middle of leaf blade, with rounded base; flower pedicels glabrous or sparsely hairy, corolla green-tinged purple or pink, corona lobes pink, pinkish or purple, hoods 3.5–5.0 mm long, with rounded apex	***Asclepiassyriaca* L.**

## Discussion

Analysis of all available information revealed that the first escape of *A.speciosa* in Lithuania was recorded in 1962 in the district of Alytus. Considering information provided on herbarium labels, this species has been recorded in two separate locations in what is now the Žuvintas Biosphere Reserve and in its vicinity, on the outskirts of the village of Liepakojai. Therefore, the currently studied colony of *A.speciosa* has existed for at least 26 years and this species can be considered as naturalized in Lithuania. Although the time of its introduction into Lithuania is unknown, we suppose that *A.speciosa* might have been introduced at the end of the 19^th^ century or the beginning of the 20^th^ century as an ornamental plant at the nearby Riečiai Manor Park (Mašalaitis 2015). It was later cultivated by local people in their gardens as an ornamental or to attract bees, and then likely escaped from cultivation.

Fruit set and sexual reproduction of *A.speciosa* were not recorded in this study. Therefore, it is possible that this population consists of vegetative descendants of one plant and represents a single clone. It is known that most species of the genus *Asclepias* are primarily or completely self-incompatible (Wyatt and Broyles 1994), and viable seeds are produced in the case of flower pollination from pollen of a genetically distinct individual. Self-compatible individuals are rare even in natural populations of some *Asclepias* species (Bookman 1984; Finer and Morgan 2003). Thus, absence of sexual reproduction significantly reduces the possibility of *A.speciosa* spreading and occupying new habitats. However, in North America, hybrids of *A.speciosa* and *A.syriaca* occur over a broad range in sympatric populations (Woodson 1954; Adams et al. 1987; Wyatt and Broyles 1994). Therefore, in situations where both species occur together in Europe, hybrids can possibly be produced, and these could start to spread.

In its native habitat of Washington State (USA), individual reproductive shoots of *Asclepiasspeciosa* produce 2–7 (mean 5) inflorescences (Finer and Morgan 2003). In the population studied in Lithuania, individual shoots produced 2–5 (mean 3.4) inflorescences. Although the number of inflorescences was lower in our studied population, the number of flowers in individual inflorescences was higher than in the native range. The number of flowers in an inflorescence in the studied population ranged from 22 to 57 (mean 36.2), whereas in Washington State the number of flowers ranges from 15 to 25 (Finer and Morgan 2003). Such differences might be influenced by climatic and ecological conditions of habitats, and may possibly represent a compensatory mechanism for lower numbers of inflorescences on each shoot. Another factor might be that the population in Lithuania is situated significantly to the north (54°28.51'N) compared to those studied by Finer and Morgan (2003) in Washington State, USA (47°25.43'N).

In its native area in North America, *A.speciosa* grows in a broad range of moisture conditions. Usually it is found in moderately wet but well-drained soil, though quite frequently it is found in riparian sites and sub-irrigated or occasionally flooded habitats. Occasionally, populations of this species can also be found in very dry sites (Stevens 2000; Young-Mathews and Eldredge 2012). Therefore, the mire habitat in which the first Lithuanian population of *A.speciosa* was found fits quite well with the range of moisture conditions found in the native area of the species. *Asclepiasspeciosa* also prefers neutral or slightly acid soils (pH 5.0–7.0 range; Stevens 2000). In its native area it usually grows in open or nearly open, well-illuminated habitats, such as pastures, meadows, wetlands with sedges and rushes, forest clearings, untilled fields, roadsides, and river and ditch banks (Young-Mathews and Eldredge 2012).

The northern limit of the native distribution of *A.speciosa* in the southern part of Western Canada (British Columbia, Alberta, Saskatchewan and Manitoba) is at approximately 50°N (Woodson 1954; Agrawal et al. 2009). Therefore, the naturalized population in Lithuania is situated almost 5° north of these native areas. Considering the broad native distribution of *A.speciosa* from southern Manitoba west to British Columbia and south to Minnesota to northwestern Texas and California (Woodson 1954; Wilbur 1976; Endress and Bruyns 2000; Agrawal et al. 2009), one can presume that this species can naturalize in the southern part of the Boreal, Continental, Atlantic, Pannonian and Steppic regions and some areas of the Mediterranean biogeographic regions of Europe (Condé et al. 2008). Potential areas of naturalization and invasion probably coincide with those of *A.syriaca* (Botta-Dukát 2008; Medvecká et al. 2012; Kelemen et al. 2016; Pergl et al. 2016). Based on the diversity of habitats occupied by *A.speciosa* in Lithuania, it can likely invade a broad range of grassland habitats, some types of wetland, many anthropogenic habitats, and become a weed of arable lands, particularly in the Pannonian and Steppic biogeographical regions of Europe. The effect of this species on habitats, their biodiversity and economic impact can be considered comparable to that of *A.syriaca*. A major obstacle to the spreading of this species is the apparent absence of sexual reproduction in the studied populations in Europe. However, further observations on the fruit setting and possible sexual reproduction of the plant in Europe are required.

## Conclusions

Currently, *A.speciosa* is a very rare alien species and Lithuania is the first documented country of occurrence in Europe. However, the possibility that this species already occurs in other regions of Europe cannot be excluded. It is possible that this species has been overlooked due to resemblance with the quite variable and similar *A.syriaca*, particularly when plants are examined at the vegetative stage. Therefore, botanists and ecologists should pay particular attention to species of the genus *Asclepias* in nature, as well as critically review herbarium specimens. It seems unlikely that *A.speciosa* has only been introduced into Lithuania.
